# Natural chalcones as dual inhibitors of HDACs and NF-κB

**DOI:** 10.3892/or.2012.1870

**Published:** 2012-06-15

**Authors:** B. ORLIKOVA, M. SCHNEKENBURGER, M. ZLOH, F. GOLAIS, M. DIEDERICH, D. TASDEMIR

**Affiliations:** 1 Laboratory of Molecular and Cellular Biology of Cancer, Cancer and Blood Research Foundation, Kirchberg Hospital, Luxembourg, Luxembourg; 2 Department of Pharmaceutical and Biological Chemistry, School of Pharmacy, University of London, London WC1N 1AX, UK; 3 Comenius University, Faculty of Sciences, Department of Microbiology and Virology, Bratislava, Slovakia

**Keywords:** chalcone, HDAC, NF-κB, dual inhibition, inflammation, cancer

## Abstract

Histone deacetylase enzymes (HDACs) are emerging as a promising biological target for cancer and inflammation. Using a fluorescence assay, we tested the *in vitro* HDAC inhibitory activity of twenty-one natural chalcones, a widespread group of natural products with well-known anti-inflammatory and antitumor effects. Since HDACs regulate the expression of the transcription factor NF-κB, we also evaluated the inhibitory potential of the compounds on NF-κB activation. Only four chalcones, isoliquiritigenin (no. 10), butein (no. 12), homobutein (no. 15) and the glycoside marein (no. 21) showed HDAC inhibitory activity with IC_50_ values of 60–190 μM, whereas a number of compounds inhibited TNFα-induced NF-κB activation with IC_50_ values in the range of 8–41 μM. Interestingly, three chalcones (nos. 10, 12 and 15) inhibited both TNFα-induced NF-κB activity and total HDAC activity of classes I, II and IV. Molecular modeling and docking studies were performed to shed light into dual activity and to draw structure-activity relationships among chalcones (nos. 1–21). To the best of our knowledge this is the first study that provides evidence for HDACs as potential drug targets for natural chalcones. The dual inhibitory potential of the selected chalcones on NF-κB and HDACs was investigated for the first time. This study demonstrates that chalcones can serve as lead compounds in the development of dual inhibitors against both targets in the treatment of inflammation and cancer.

## Introduction

Acetylation is a pivotal post-transcriptional modification, which strongly influences chromatin structure and function (1). Due to its wide variety of targets, it is not only implicated in the regulation of gene expression via chromatin structure modifications but also in protein-protein interactions, protein stability, DNA binding, and subcellular localization (2). Histone acetylation is mediated by histone acetyltransferases (HATs). The resulting structural modification of chromatin leads to nucleosomal relaxation and altered transcriptional activation. The reverse reaction is mediated by histone deacetylases (HDACs), which induce deacetylation, chromatin condensation, and transcriptional repression (3). Alterations mediated by HAT/HDAC activities are not solely reduced to chromatin. Mounting evidence accentuates their involvement in lysine acetylation/deacetylation of non-histone substrates including transcription factors (such as NF-κB, p53, GATA2, MEF2) and chromatin-associated co-repressor proteins (4,5). A balanced histone acetylation status is essential for the proper progress of cell proliferation, apoptosis and differentiation. An improper HDAC recruitment or activity, often leads to abnormal gene expression that is associated with cancer development (6), hence rendering HDAC as an excellent target in current cancer research. Two HDAC inhibitors, suberoylanilide hydroxamic acid (SAHA; vorinostat) and the natural product romidepsin (FK-228) are currently approved for cancer chemotherapy and many other inhibitors are in clinical trials (7). So far, eighteen human HDAC enzymes have been identified and grouped into four classes based on the structure of their accessory domains. Classes I, II, and IV, but not III, require a zinc molecule as an essential cofactor in their active site and are inhibited by Zn^2+^-binding HDAC inhibitors such as SAHA and the natural product, trichostatin A. Class I HDACs comprise HDACs 1, 2, 3 and 8, whereas class II HDACs include HDACs 4, 5, 6, 7 and 9 that are larger in size than the other classes (4,5). Recent publication of the X-ray crystal structure of HDAC8 (8) followed by several other HDACs (7) fuelled the research activity in the discovery and development of novel HDAC inhibitors.

The transcription factor NF-κB is a dimer of proteins belonging to the Rel family. It is an ubiquitous transcription factor present in all cell types. The most common form of NF-κB is the p65/p50 heterodimer. In most cells, NF-κB complexes are localized to the cytosol as inactive forms with the inhibitor of κB protein (IκB). Phosphorylation of IκB results in its ubiquitination and subsequent proteasome-mediated degradation. Activated NF-κB then translocates to the nucleus where it transactivates more than 500 target genes (9). Many factors are known to activate NF-κB, including inflammatory cytokines such as tumor necrosis factor alpha (TNFα) and interleukin (IL)-1, carcinogens (cigarette smoke), UV radiation, hyperglycemia and tumor promoters. Over the last decade, NF-κB became a major target in drug discovery, due to its key role in cancer development, inflammation, cell proliferation and death (10).

Recent evidence indicates that the activation of various transcription factors, including NF-κB, is regulated through the interaction with HDAC proteins (11–13). Previous studies with various cell types showed that HDAC1 and HDAC2 negatively regulate NF-κB activity through direct interaction with the p65 (RelA) subunit of NF-κB (14,15). Other studies suggest a physical interaction between p65 (RelA) and class I type HDACs (HDAC1, 2 and 3), where high expression of class I HDACs has been linked to increased nuclear translocation of p65 (RelA) (16). Remarkably, Class I HDAC isoforms are often overexpressed in various types of cancers where they are usually associated with a poor prognosis (5,17,18). In addition, HDAC inhibitors are acknowledged as effective anti-inflammatory agents, some inhibiting NF-κB (19–21) and may therefore play an important role in the prevention of cancers that develop as a result of chronic inflammation. The combined antiproliferative as well as anti-inflammatory potency represents a highly attractive combination for the treatment of numerous chronic inflammatory conditions which are frequently associated with an increased risk of developing cancer.

Chalcones (1,3-diphenyl-2-propenones) are a group of aromatic compounds that represent a large class of natural products found in many medicinal plants, fruits, vegetables, spices and nuts. They are the natural precursors of flavonoids and display a variety of biological activities. Although the modes of action of this class of compounds are not fully understood, great efforts are devoted to elucidate the mechanisms underlying their promising anti-inflammatory and anticancer activities. Hence several natural chalcones have been reported to inhibit the NF-κB signaling, and numerous synthetic derivatives have been evaluated in structure-activity relationship (SAR) studies (22,23). Besides the NF-κB inhibition, interference in microtubule formation is generally thought to be responsible for their anticancer activities (24,25). Despite the cross-talk and modulation effects between NF-κB and HDACs, and structural similarity of chalcones to broad-spectrum HDAC inhibitors SAHA and trichostatin A, HDACs have not been investigated as potential targets for natural chalcones. In this study, we aimed to test twenty-one commercially available chalcones (Table I) for dual HDACs and NF-κB inhibitory activities *in vitro*. Viability assays were also carried out to elucidate the cytotoxic potential of the chalcones against leukemia cells. We also aimed to explore SAR to determine the essential functionalities on the chalcone core for biological activity. We also performed molecular modeling and docking studies in an attempt to understand the potential mode and mode/site of binding of natural chalcones to NF-κB and class I type HDACs.

## Materials and methods

### Chalcones

Twenty-one natural chalcones, namely chalcone, 2′-hydroxychalcone, 2-hydroxychalcone, 4-hydroxychalcone, 4-methoxychalcone, 3,4-dimethoxychalcone, 4′-hydroxychalcone, 4′-methoxychalcone, 4,4′-dimethoxychalcone, isoliquiritigenin (2′,4,4′-trihydroxychalcone), calomelanone (2′,6′-dihydroxy-4,4′-dimethoxydihydrochalcone), butein (2′,3–4,4′-tetrahydroxychalcone), flavokawain C (2′,4-dihydroxy-4′,6′-dimethoxychalcone), gymnogrammene (2′,6′-dihydroxy-4,4′-dimethoxychalcone), homobutein (2′,4,4′-trihydroxy-3-methoxychalcone), 2,3-dimethoxy-2′-hydroxychalcone, flavokawain A (2′-hydroxy-4,4′,6′-trimethoxychalcone), eriodictyolchalcone (2′,4′,6′,3,4-pentahydroxychalcone) phloretin (2′,4,4′,6′-tetrahydroxydihydrochalcone), phloridzin (phloretin-2′-O-glucoside) and marein (2′,3,3′,4,4′-pentahydroxy-4′-glucosylchalcone) were studied. Table 1 shows their chemical features and substitution patterns. Chalcone was purchased from Fluka (Steinheim, Germany), whereas all remaining chalcones were obtained from Extrasynthese (Genay Cedex, France) (purity >97%).

### Cell culture and reagents

Human Philadelphia chromosome-positive chronic myelogenous leukemia cell line K562 was purchased from Deutsche Sammlung für Mikroorganismen und Zellkulturen (DSMZ, Braunschweig, Germany) and cultured in RPMI-1640 medium (Lonza, Verviers, Belgium) supplemented with 10% fetal calf serum (Hyclone, Perbio, Erembodegem, Belgium) and 1% (v/v) antibiotic-antimycotic (Lonza, BioWhittaker™), at 37°C, in a 5% CO_2_, humidified atmosphere. Human recombinant TNFα (PeproTech, Rocky Hill, NJ, USA) was resuspended in 1× phosphate-buffered saline (PBS) sterile solution containing 0.5% bovine serum albumin (MP Biomedicals, Asse-Relegem, Belgium) to reach a final concentration of 10 μg/ml.

### HDAC activity/inhibition measurement

Direct HDAC inhibition by chalcone derivatives was estimated using K562 total extracts as an HDAC source and the enzymatic HDAC activity measurement was performed using a fluorometric HDAC assay kit (Active Motif, Rixensart, Belgium) according to the manufacturer’s instructions. Briefly, after being washed twice with ice-cold 1× PBS, cells were pelleted by centrifugation, and lysed in M-PER^®^ mammalian protein extraction reagent (Pierce, Erembodegem, Belgium) and supplemented with 1× protease inhibitor cocktail (Roche, Prophac, Luxembourg). The cell suspension was gently mixed on an orbital shaker for 15 min and centrifuged at 14,000 × g at 4°C for 15 min. Protein content was assessed using the Bradford assay (Bio-Rad, Nazareth, Belgium), and 10 μg of proteins were incubated with vehicle or various concentrations of the different chalcones for 1 h at 37°C in the presence of an HDAC fluorometric substrate. Subsequently, the HDAC assay developing solution was added and after 15 min of incubation at room temperature, the fluorescence was measured using a Gemini EM microplate spectrofluorometer (Molecular Devices, Belgium) with excitation at 360 nm and emission at 460 nm. The measured activities were normalized by the vehicle-treated control enzyme activities and IC_50_ values were calculated.

### Transient transfections

Transient transfections of K562 cells were performed as previously described (26).

### In vitro cytotoxicity assays (viability assay)

The *in vitro* growth inhibitory values of chalcone derivatives on the K562 cell line were determined as detailed previously (26).

### Molecular modeling and docking studies

The 2D structures of chalcone molecules were drawn using SketchEI and transferred into the VEGA ZZ molecular modeling software (27,28) to generate 3D structures. All molecules were saved into a single mol file, that was used as input for the OMEGA, OpenEye Scientific Software (Omega version 2.3.2; http://www.openeye.com) to generate a maximum of 2 low energy conformers with default values. These conformations were stored as OEB file extension format and their 3D similarity was compared using the Rocs, OpenEye Scientific Software (Rocs version 2.3.1; http://www.openeye.com). E-Dragon Software (29) was utilized to calculate constitutional and molecular property descriptors. The descriptors selected to describe the SAR were selected using Partial Least Squares regression as implemented in the PLSR module of Virtual Computational Chemistry Laboratory (29) and Gretl software was used to calculate the correlation between the logarithm of the activity and predicted molecular properties.

The molecular docking was carried out using Glide software (Grid-Based Ligand Docking With Energetics) (Schrodinger Inc., Portland, OR, USA) (30,31) after the docking targets were prepared using Protein Preparation Wizard workflow in Maestro (Schrodinger Inc.) by removing water molecules, adding the hydrogen atoms and assigning all atom force field (OPSL-2005) charges and atom types. The position of all atoms was adjusted by minimizations until the average root mean square deviation reached 0.3 Å. The crystal structures of HDAC8 wild-type and variant D101 complexed with ligands [Protein Data Bank (pdb) entries 1T69 and 3EZT] were used for molecular docking of chalcones into the protein active site. The box encompassing the active site was selected based on the position of co-crystalized ligands complex as described in a previous study (32). The crystal structure of NF-κB complexed to DNA was chosen as a target system to elucidate binding modes of chalcones (pdb entry 1NFK). Prior to docking the DNA molecule was removed and the coordinates of the enclosing box of 30 Å (center at × = −1,1958 Å; y = 9.0149 Å; z = 19,7598 Å) were encompassing the active site residues involved in hydrogen bonds with the NF-κB recognition site of DNA (Arg54, Arg56, Tyr57, Cys59, Lys241, Gln306 and Thr143) (35). Flexible ligand docking simulations were carried out with Glide using the default settings. The ten best poses obtained using the Extra-Precision Glide (Glide XP) mode were selected for analysis. The most favorable poses of molecules showing activity were subjected to further energy minimization using Macromodel 9.1 and OPLS2005 force field.

## Results

### Inhibition of HDAC activity by chalcone derivatives

**Figure 1:**
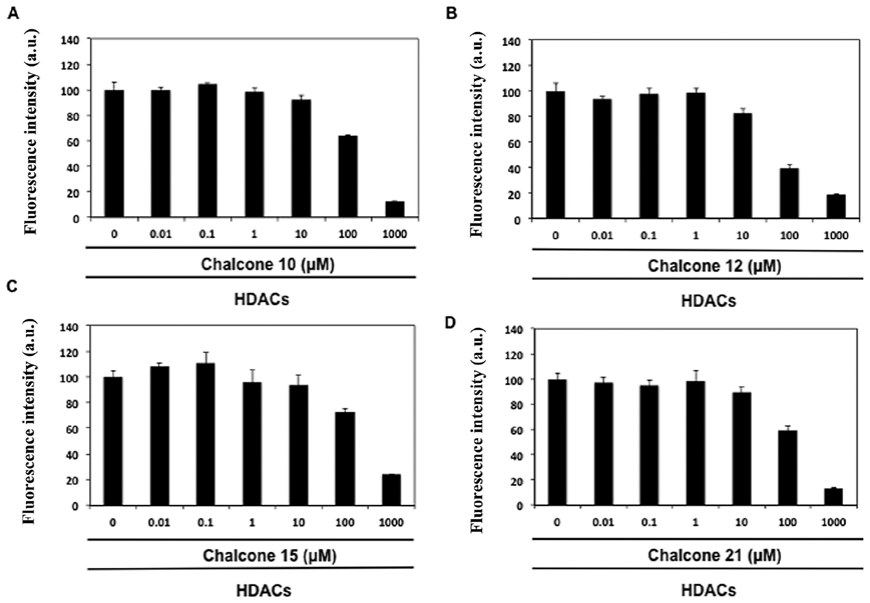
Inhibition of HDAC activity by active chalcone derivatives. Total protein extracts from K562 cells were incubated with vehicle (0) or various concentrations of the (A) chalcone 10 (B) chalcone 12 (C) chalcone 15 or (D) chalcone 21 for 1 h in the presence of an HDAC fluorometric substrate. Fluorescence was measured using a Gemini EM microplate spectrofluorometer and normalized by the vehicle-treated control enzyme activities. Vehicle-treated control corresponds to 0 on the chart. Results are presented as a mean ± SD of at least three independent experiments.

The effect of chalcone derivatives (nos. 1–21) was examined on total HDAC activity using a fluorescence HDAC assay. As shown in Table II, four chalcone aglycones, namely isoliquiritigenin (no. 10), butein (no. 12), homobutein (no. 15) and the glycoside marein (no. 21), reduced HDAC activity in a concentration-dependent manner (IC_50_ values 60–190 μM, Fig. 1). Butein (no. 12) appeared to be the best inhibitor of HDAC activity. Other chalcone derivatives were assumed as inactive, because they were unable to provide distinct inhibitory effect even at the highest test concentration (1000 μM).

### Inhibition of TNFα-induced NF-κB activation by chalcones

**Figure 2:**
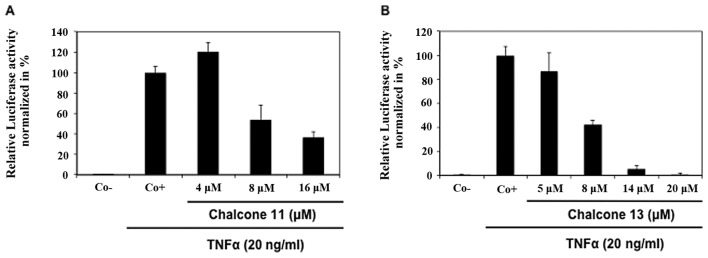
Inhibition of TNFα-induced NF-κB activation by (A) chalcone 11 and (B) chalcone 13. K562 cells were transiently transfected with firefly luciferase vector (NF-κB pGL4), and ph-RG-tk Renilla plasmid for 24 h. After transfection, K562 cells were treated with chalcone derivatives at the different concentrations for 2 h followed by a TNFα-treatment (20 ng/ml) during 6 h. Results are expressed as a ratio of the measured luminescence of the firefly luciferase vector and the luminescence of Renilla plasmid. Results are presented as a mean ± SD of three independent experiments. Negative control (Co−) corresponds to DMSO treated cells, without TNFα activation, positive control (Co+) corresponds to DMSO treated cells activated by TNFα.

By using a luciferase-based *in cellulo* NF-κB reporter assay, chalcones were evaluated for TNFα-induced NF-κB transcription inhibition activity (Table II). Flavokawain C (no. 13) was the most potent NF-κB inhibitor, followed by the dihydrochalcone calomelanone (no. 11) with IC_50_ values of 8 and 11 μM, respectively (Fig. 2). Chalcones 4-hydroxychalcone (no. 4), 4-methoxychalcone (no. 5), 4′-hydroxychalcone (no. 7), isoliquiritigenin (no. 10), butein (no. 12), homobutein (no. 15) and phloretin (no. 19) also demonstrated good potential with IC_50_ values ranging between 24–41 μM. Three polymethoxychalcones, i.e. 4,4′-dimethoxychalcone (no. 9), gymnogrammene (no. 14), flavokawain A (no. 17), as well as cone eriodictyolchalcone (no. 18) and two chalcone glycosides, phloridzin (no. 20) and marein (no. 21), which showed no or limited inhibitory activity at 200 μM concentration, were considered as inactive. The remaining compounds, chalcone (no. 1), 2′-hydroxychalcone (no. 2), 2-hydroxychalcone (no. 3), 3,4-dimethoxychalcone (no. 6), 4′-methoxychalcone (no. 8) and 2,3-dimethoxy-2′-hydroxychalcone (16) were cytotoxic at concentrations, which inhibited NF-κB activation.

### Docking studies of chalcones 12 and chalcone 21 within the active site of HDAC8

**Figure 3:**
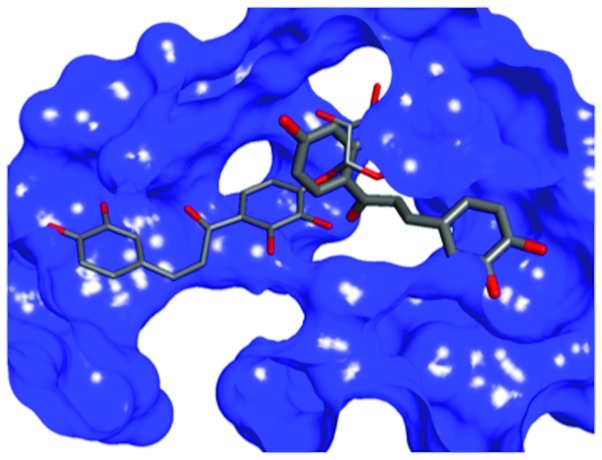
Best binding poses of chalcone 12 (thick stick representation) and chalcone 21 (thin stick representation) within the active site of the HDAC8 represented by surface only. The docking was carried out using Glide and DS Visualizer 3.1 was used to prepare the image.

In order to shed light on the potential mode of action of chalcones, and to understand why some chalcones inhibit either NF-κB or HDACs and some inhibit both, we have carried out molecular modeling, molecular similarity and docking studies. The compounds were docked into the binding sites of HDAC8, and the best studied HDAC enzyme was selected based on the position of the co-crystalized ligand in the crystal structure of the complex (pdb entries 1T69 and 3ETZ) (32). The GlideScore values were compared to the activities that were experimentally obtained (Table III). The results of the docking indicated that all chalcones could favorably bind in the active site, although not all molecules showed activity. The most active molecule 12 had a less favorable GlideScore than chalcone 21 that exhibited the most favorable binding. The binding mode of these two molecules is different (Fig. 3) which may be the result of the large active site of HDAC8 which accommodates two (4-(dimethylamino)-N-[7-(hydroxyamino)-7-oxoheptyl]benzamide) moieties in the interior pocket of the protein. Furthermore, the docking could not distinguish the third active molecule 10 from the rest of the group. There is a clear difference between the GlideScore of the binding of 21 to 20. However, 20 binds almost as good as 12 and better than 10, resulting in the absence of the correlation between activity and binding affinity determined by Glide. We hypothesize that molecules that are not active could possibly bind preferably elsewhere on the protein surface rather than on the active site.

The docking studies of chalcones were also carried out using a crystal structure of NF-κB dimer in complex with duplex DNA. We followed a procedure reported by Piccagli *et al*(33) and have chosen the DNA recognition surface to define the docking target. The binding site included residues Arg54, Arg56, Tyr57, Cys59, Lys241, Gln306 and Thr143 to gain information on the interaction of our compounds with NF-κB. As shown in Table III, there is a lack of correlation between the activity and the GlideScore results. This can be due to non-specific binding. Moreover, chalcones could act on a different active site of NF-κB. To further rationalize the activity of this class of compounds we have elucidated the SARs and predicted more than 1600 molecular properties for all molecules using EDragon software (29). This analysis was not aimed to lead to the development of predictive models since the data set is small (fifteen molecules with measured NF-κB activity) and thus we did not create training and test sets. Two different sets of molecules were subjected to partial least square regression using PLSR module of the Virtual Computational Chemistry Laboratory to determine which constitutional and molecular properties correlated with the negative logarithm of activity (pNF-κB). The first group consisted of all fifteen tested molecules and the observed correlation was not satisfactory (r^2^=0.53), leading us to examine the second smaller group, consisting of only nine molecules that displayed activity. The PLS results indicated that a combination of nineteen descriptors correlated with activity (r^2^=0.99). There were some highly correlated and irrelevant descriptors selected to describe correlation. The selection of descriptors was optimized by developing least square methods using the Gretl software and removing descriptors until a minimal number of descriptors was obtained with a good correlation (r^2^= 0.914, s=0.250, n=9, F=3.48). The formula used was the following: pNF-κB = −79.78(±48.55) − 0.425(±.0.344)*SS + 31.582(±24.)^*^Mp + 43.847(±25.978)^*^ARR + 1.868 (±1.619)^*^Hy + 0.265(±.468)^*^MLOGP + 2.793 (±1.682)^*^nBO.

The list and values of descriptors, observed and calculated are shown in Table IV. The statistics indicate reasonable descriptive value of the model that shows which molecular properties influence activity of the molecules in the NF-κB assay. Since we could not develop a satisfactory model that would differentiate the active and inactive molecules, we examined the molecular similarity and differences between most active molecules and the inactive ones. Conformational search carried out by Omega software and the Merck Molecular Force Field force revealed that all molecules can exist in several different conformations due to the free rotation around bonds between carbonyl carbon and neighboring groups. ROCS search and comparison of electrostatic forces showed that unsurprisingly molecules are similar (Shape Tanimoto coefficients are between 0.94 and 0.0.69 and Tverstsky coefficients are between 0.91 and 0.71). The highest similarity was observed between the most active chalcone molecule 13 and inactive 9, indicating that shape and electrostatic properties are not sufficient to explain the different activities of the group.

## Discussion

Chronic inflammation has been linked to most incurable illnesses, including cancer, cardiovascular and neurodegenerative diseases. Cancer is regulated by a large number of genes that are modulated by transcription factors, such as NF-κB, which controls genes involved in inflammation, proliferation, angiogenesis and metastasis (23). Any disturbance in the corresponding pathways leads to the activation of NF-κB and release of cytokines, thus contributing to the initiation and progression of tumorigenesis. On the other hand, acetylation and deacetylation act as regulating mechanisms for activation or inactivation of various transcription factors, including NF-κB. This process is mediated by HDAC and can consequently be modulated by HDAC inhibitors (34). Protein complexes involved in the regulation of cell-cycle progression and apoptosis are also controlled by this mechanism (2). The reversible acetylation appears to regulate the interaction between p65 and IκB, and controls the duration of the NF-κB response. NF-κB activation leads to apoptosis resistance. Several NF-κB inhibitors showed potential to overcome this resistance and induce apoptosis (35,36). Thus, inhibition of NF-κB may sensitize cancer cells and eventually lead to the induction of apoptosis. Interestingly, in our study, chalcones 4, 5, 7, 10, 11, 12, 13, 15 and 19 inhibited both NF-κB and the viability of K562 cells.

Interestingly, three chalcones (nos. 10, 12, and 15) inhibited both NF-κB and HDAC activity. Nevertheless, underlying mechanisms in the action of chalcones as dual inhibitors remain to be elucidated in the future. To our knowledge, the mechanisms that link both inhibitory activities were first reported herein. As an example, upon interaction with histone deacetylase 3 (HDAC3), p65 is deacetylated leading to efficient interaction with IκB and subsequent activation via the canonical pathway (37). Compounds that efficiently inhibit HDAC3 and other HDAC isoforms are considered interesting NF-κB inhibitors. Moreover, transcription factor STAT1 is physiologically acetylated and binds p65, thus inhibiting NF-κB. STAT1-associated HDAC were described to deacetylate STAT1 leading to the liberation of p65 and subsequent activation of the canonical NF-κB pathway. Inhibitors of HDAC activity contribute to a shift towards acetylated STAT1 actively interacting with NF-κB and thus inhibiting activation of p65 required for inflammatory cell signaling (38). Even though the inhibitory activity of selected chalcones appears weak when only the total HDAC activity is assessed, inhibitory effect against specific HDAC isoforms are generally stronger as shown for tubastatin A (39).

Attempts to generate HDAC inhibitors generally focus on varying the cap group to exploit variability in the HDAC surface surrounding the active site. However, although efforts are being made to identify truly class- or isoform-selective HDAC inhibitors with anticancer and anti-inflammatory properties, the current list of such compounds remains relatively poor. One of the main reasons is the lack of structural determinants of selective HDAC inhibition due to the challenge of studying the interaction of small inhibitory molecules with multiple large protein complexes that encompass HDAC activities, which are often dependent on or regulated by these complexes. Accordingly, the selectivity of small inhibitory molecules may depend on the context of HDAC complexes and requires investigation (40). The small size of the library limits the ability to obtain common structural features necessary for individual or dual target activity.

A number of SAR studies have been performed on synthetic chalcones and their biological effects, including NF-κB inhibition (22,23). To our knowledge this is the first study looking at potential SARs among natural chalcones. In the present study, some chalcones showed interesting NF-κB inhibitory potential, and some empirical SARs have been obtained. Our results show that chalcone, the parent compound, or its 2′-hydroxy- or 2-hydroxy- derivatives (nos. 1–3) did not express any NF-κB inhibition potential. We were able to draw some SARs originating from the substitutions with an electron donating functional group such as methoxy or hydroxy, at positions 4, 4′ and 6′. Chalcone glycosides (nos. 20 and 21) were not active, which might be due to the reduced permeability through cell membranes. In addition, all chalcones with reported NF-κB activity contain a highly electrophilic α,β-unsaturated carbonyl moiety and calomelanone and phloretin are the first examples of dihydrochalcones with such activity. For HDAC activity clearer trends were observed, i.e. hydroxy groups at C-2′, C-4′, C-3 and C-4 were essential. This substitution pattern also appears to be important for dual activity. The empirical SARs prompted us to perform some molecular modeling-docking studies on both targets. The molecular modeling investigations could not provide a definite rationale for dual activity of chalcones and failed to provide criteria for distinguishing the active molecules from inactive. There could be many reasons underlying this observation. Most of the molecules have low molecular weight (between 200 to 275 Da) and as such can be considered as fragments. This could lead to non-specific binding and lack of correlation between molecular properties and activity.

Some of the compounds tested herein have previously been reported as NF-κB inhibitors including isoliquiritigenin (no. 10) (41) and butein (no. 12) (42). We have previously demonstrated that 4′-hydroxychalcone showed 26S protease inhibition activity on three different proteolytic activities (chymotrypsin, trypsin- and caspase-like) in a dose-dependent manner (26). The involvement of natural chalcones in cancer tumorigenesis has been reviewed (43). To the best of our knowledge this is the first study aiming to screen a natural chalcone library and attempting to draw SARs among them. Several natural chalcones emerged as relatively good inhibitors of NF-κB. Additionally, HDAC was identified as a novel potential target for the chalcones described.

## Figures and Tables

**Table I:** Structural features of natural chalcones.

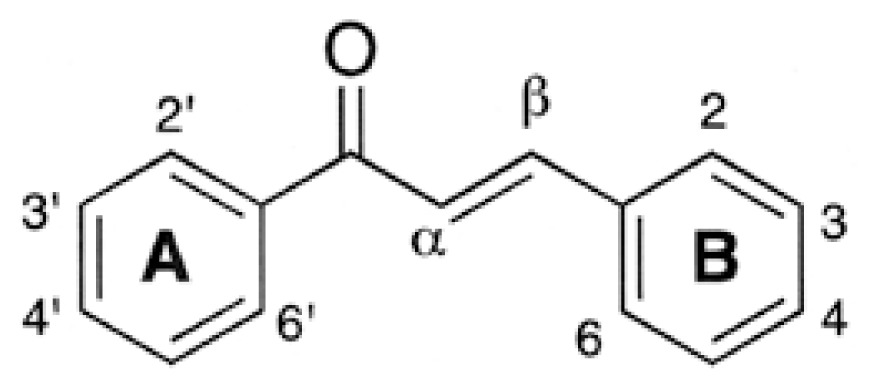								

	Substitution							
	
Chalcone	2′	3′	4′	6′	2	3	4	Δα,β
Chalcone, no.1	H	H	H	H	H	H	H	Unsaturated
2′-Hydroxychalcone, no 2	OH	H	H	H	H	H	H	Unsaturated
2-Hydroxychalcone, no 3	H	H	H	H	OH	H	H	Unsaturated
4-Hydroxychalcone, no 4	H	H	H	H	H	H	OH	Unsaturated
4-Methoxychalcone, no 5	H	H	H	H	H	H	OCH3	Unsaturated
3,4-Dimethoxychalcone, no 6	H	H	H	H	H	OCH3	OCH3	Unsaturated
4′-Hydroxychalcone, no. 7	H	H	OH	H	H	H	H	Unsaturated
4′-Methoxychalcone, no 8	H	H	OCH3	H	H	H	H	Unsaturated
4,4′-Dimethoxychalcone, no 9	H	H	OCH3	H	H	H	OCH3	Unsaturated
Isoliquiritigenin (2′,4,4′-trihydroxychalcone), no 10	OH	H	OH	H	H	H	OH	Unsaturated
Calomelanone (2′,6′-dihydroxy-4,4′-dimetoxydihydrochalcone), no 11	OH	H	OCH3	OH	H	H	OCH3	Saturated
Butein (2′,3–4,4′-tetrahydroxychalcone), no 12	OH	H	OH	H	H	OH	OH	Unsaturated
Flavokawain C (2′,4-dihydroxy-4′,6′-dimethoxychalcone), no 13	OH	H	OCH3	OCH3	H	H	OH	Unsaturated
Gymnogrammene (2′,6′-dihydroxy-4,4′-dimethoxychalcone), no 14	OH	H	OCH3	OH	H	H	OCH3	Unsaturated
Homobutein (2′,4,4′-trihydroxy-3-methoxychalcone), no 15	OH	H	OH	H	H	OCH3	OH	Unsaturated
2,3-Dimethoxy-2′-hydroxychalcone, no 16	OH	H	H	H	OCH3	OCH3	H	Unsaturated
Flavokawain A (2′-hydroxy-4,4′,6′-trimethoxychalcone), no 17	OH	H	OCH3	OCH3	H	H	OCH3	Unsaturated
Eriodictyolchalcone (2′,4′,6′,3,4-pentahydroxychalcone), no 18	OH	H	OH	OH	H	OH	OH	Unsaturated
Phloretin (2′,4,4′,6′-tetrahydroxydihydrochalcone), no 19	OH	H	OH	OH	H	H	OH	Saturated
Phloridzin (phloretin-2′-O-glucoside), no 20	O-Glu	H	OH	OH	H	H	OH	Saturated
Marein (2′,3,3′,4,4′-pentahydroxy-4′-glucosylchalcone), no 21	OH	OH	O-Glu	OH	H	OH	OH	Unsaturated

Glu, glucose.								

**Table II:** Biological activity of natural chalcones.

Chalcone	HDAC inhibition, IC_50_ (μM)	NF-κB inhibition, IC_50_ (μM)	Viability
1	>1000	n.m.	14
2	>1000	n.m.	28
3	>1000	n.m.	2
4	>1000	24	31
5	>1000	29	35
6	>1000	n.m.	38
7	>1000	28	16
8	>1000	n.m.	38
9	>1000	>200	n.t.
10	110	32	44
11	>1000	11	31
12	60	38	13
13	>1000	8	13
14	>1000	>200	n.t.
15	190	38	29
16	>1000	n.m.	12
17	>1000	>200	n.t.
18	>1000	>200	n.t.
19	>1000	41	59
20	>1000	>200	n.t.
21	100	>200	n.t.
Standards	0.14^b^	6.0^a^	1.4^a^

n.m., not measurable due to toxicity after transfection; n.t., not tested due to the inability to inhibit the activation of NF-κB; Standards, ^a^heteronemin, ^b^suberoylanilide hydroxamic acid.

**Table III:** GlideScore values obtained for HDAC and NF-κB proteins.

Chalcone	Docking scores against 1T69	Docking scores against 3ETZ	Docking scores against 1NFK
1	−6.83	−5.27	−2.39
2	−5.90	−6.08	−4.05
3	−5.40	−6.35	−3.06
4	−4.71	−6.27	−2.38
5	−5.52	−5.34	−3.22
6	−4.92	−5.87	−2.76
7	−4.88	−5.96	−2.88
8	−5.78	−5.86	−2.48
9	−5.88	−5.70	−2.6
10	−6.03	−7.03	−2.84
11	−6.75	−7.21	−3.15
12	−7.00	−8.00	−4.71
13	−5.32	−7.45	−3.48
14	−5.63	−6.52	−4.29
15	−6.44	−7.06	−0.71
16	−5.31	−6.57	−3.1
17	−4.47	−6.79	−1.75
18	−5.69	−7.24	−4.06
19	−5.15	−6.47	0.12
20	−6.85	−7.57	−5.53
21	−8.08	−10.51	−6.03

**Table IV:** Predicted molecular properties correlating to the activity of potent chalcones in the NF-κB assay.

Chalcone	Ss	Mp	nBO	Hy	MLOGP	ARR	Observed pNF-κB	Calculated pNF-κB
4	41.67	0.71	18	−0.371	3.148	0.667	4.62	4.59
5	41.17	0.69	19	−0.873	3.398	0.632	4.54	4.56
7	41.67	0.71	18	−0.371	3.148	0.667	4.55	4.59
10	53.00	0.69	20	1.096	2.552	0.600	4.49	4.37
11	56.67	0.65	23	0.304	2.334	0.522	4.96	4.97
12	58.67	0.68	21	1.928	1.764	0.571	4.42	4.51
13	57.67	0.67	23	0.304	1.746	0.522	5.10	5.02
15	58.17	0.67	22	0.545	1.062	2.013	4.42	4.51
19	58.67	0.68	21	1.928	1.764	0.571	4.39	4.40

Ss, Sum of Kier-Hall electrotopological states; Mp, mean atomic polarizability (scaled on Carbon atom); nBO, number of non-H bonds; Hy, hydrophilic factor; MLOGP, Moriguchi octanol-water partition coefficient (logP); ARR, aromatic ratio; pNF-κB, negative logarithm values of activity observed in NF-κB assay.
